# Cultural effects on computational metrics of spatial and temporal context

**DOI:** 10.1038/s41598-018-20200-y

**Published:** 2018-02-01

**Authors:** Nicholas D. Wright, Jan Grohn, Chen Song, Geraint Rees, Rebecca P. Lawson

**Affiliations:** 1 Institute for Conflict, Cooperation and Security, University of Birmingham, Birmingham, UK; 2 Department of Experimental Psychology, University of Oxford, Oxford, UK; 3 Department of Psychiatry, University of Wisconsin-Madison, Madison, USA; 4 Cardiff University Brain Research Imaging Centre, School of Psychology, Cardiff University, Cardiff, UK; 5 Institute of Cognitive Neuroscience, University College London, London, UK; 6 Wellcome Trust Centre for Neuroimaging, University College London, London, UK; 7 Department of Psychology, University of Cambridge, Cambridge, UK

## Abstract

The concept of “prediction error” - the difference between what occurred and was expected - is key to understanding the cognitive processes of human decision making. Expectations have to be learned so the concept of prediction error critically depends on context, specifically the *temporal* context of probabilistically related events and their changes across time (i.e. volatility). While past research suggests context differently affects some cognitive processes in East Asian and Western individuals, it is currently unknown whether this extends to computationally-grounded measures of learning and prediction error. Here we compared Chinese and British nationals in an associative learning task that quantifies behavioural effects of prediction error, and—through a hierarchical Bayesian learning model—also captures how individuals learn about probabilistic relationships and their volatility. For comparison, we also administered a psychophysical task, the tilt illusion, to assess cultural differences in susceptibility to *spatial* context. We found no cultural differences in the effect of spatial context on perception. In the domain of temporal context there was no effect of culture on sensitivity to prediction error, or learning about volatility, but some suggestion that Chinese individuals may learn more readily about probabilistic relationships.

## Introduction

Neuroscience has made substantial advances in understanding the processes by which humans perceive and respond to the environment. Importantly, using computational approaches to model and quantify such cognitive processes allows an *objective* assessment of otherwise *subjective* reports, and provides unified explanations of diverse behavioural phenomena^1–5^. Central to understanding perception and decision making is the concept of “prediction error”—the difference between what occurred and was expected^6,7^. Previous cross-cultural studies employing *subjective* reports of prediction error show that East Asians are less susceptible to prediction errors than Westerners. For instance, South Koreans report less surprise than Americans after unexpected outcomes in vignettes^8^; and in the U.S., people born in East Asia report less pleasure and surprise at an unexpected gift than those U.S. born^9^. Whether these subjective reports of cross-cultural differences also apply to the objectively measured behavioural and computational aspects of prediction errors is unknown. Here we employed computationally-grounded measures to assess susceptibility to prediction error and characterise any cross-cultural differences.

A prominent idea in the cross-cultural literature is that people from East Asia (mostly referring to people from China, Japan, or Korea) think more holistically than people from the West (mostly referring to European North Americas)^10,11^. At the heart of this distinction is a difference in how context is incorporated into perceiving and responding to the environment. East Asians tend to show *more* context-dependence than Westerners across diverse cognitive domains^10^. This ranges from being more sensitive to what appears behind or around a stimulus (i.e. spatial context)^12,13^, to having a greater sense of dependency between two temporally separated events (i.e. temporal context)^14^. Furthermore, many psychological studies indicate that people from East Asian cultures, being more holistic “big picture” thinkers, are more inclined to expect change to occur across time, and therefore show more tolerance of contradiction (see^15^ for a review), which aligns with subjective reports of diminished surprise^8,9^. Expectations have to be learned across time, and so the concept of prediction error critically depends on temporal context.

Previous research suggesting that Chinese participants are better calibrated to the probabilistic association between two temporally dissociated stimuli^14^, and also update their beliefs more readily on the basis of feedback^16^, could be consistent with faster learning of probabilistic relationships in the environment. Additionally, the rate at which probabilistic relationships change over time (i.e. volatility) is a form of temporal context that humans learn about in order to determine *when* a prediction error indicates that prior beliefs should be revised^17–22^ (e.g. faster belief updating when conditions are estimated to be volatile). Work suggesting that Chinese participants are more likely than Western participants to predict that previous conditions will reverse^23^, could reflect a greater expectation for volatility or a general tendency to better tolerate changes in volatility. Despite the fact there are established computational models that capture how humans learn to build expectations under conditions of environmental change^17–22^, no studies have examined cross-cultural differences in these mechanisms computationally.

To assess whether cultural differences in context dependence extend to these temporal domains, we employed an established probabilistic associative learning paradigm that assays the behavioural effects of prediction error as a function of learned expectations. Furthermore, we applied a hierarchical Bayesian learning model to capture individual differences in learning across trials^17–21^. To interrogate different aspects of temporal context in both Chinese and British participants we compared (1) behavioural metrics of prediction error (averaged across all trials), and computational measures of (2) learning about probabilistic relationships and (3) learning about the rate at which these relationships change (i.e. volatility). We hypothesised that objective measures of behavioural prediction error would support previous work using subjective reports^8,9^, and Chinese participants would show reduced effects of prediction error on reaction times (RT). This would correspond to a faster RT to respond to unexpected stimuli in our task. Second, we predicted that learning rates from the computational model will indicate that Chinese learn more readily than Westerners about probabilistic associations, consistent with work indicating greater sensitivity to covariation^14^. Lastly, if Chinese participants anticipate greater rates of environmental change, we predicted an effect of culture on volatility learning. This would manifest as either a greater volatility learning rate, reduced learning rate update in response to volatility (indicating greater tolerance of change) or cultural differences in the effect of volatility on behaviour (RTs). The hierarchical Bayesian model adopted here permits testing of each prediction.

Finally, we note that it is poorly understood whether cultural impacts on an individuals’ degree of context dependence generalises across multiple domains. Thus, here we also extend the cross-cultural literature on *spatial* context effects in visual perception by assaying the same participants’ context dependence in an established and psychophysically characterised ‘tilt illusion’ task. We hypothesised that culture will affect the magnitude of the spatial context effect in this perceptual task, and if individual differences in spatial and temporal context dependence are domain general then the impact of temporal context dependence in the probabilistic associative learning task may predict the effect of spatial context in the tilt illusion task.

## Results

### Temporal context: behavioural prediction errors

We used a common probabilistic associative learning (PAL) task^17,20,21^ to test the impact of violated expectations (i.e. prediction errors) on reaction times (RT)^21^ in Chinese nationals (n = 36) and age, gender, and IQ matched British nationals (n = 36) (see methods for full participant details and *a priori* power analysis to determine sample size).

**Figure 1:**
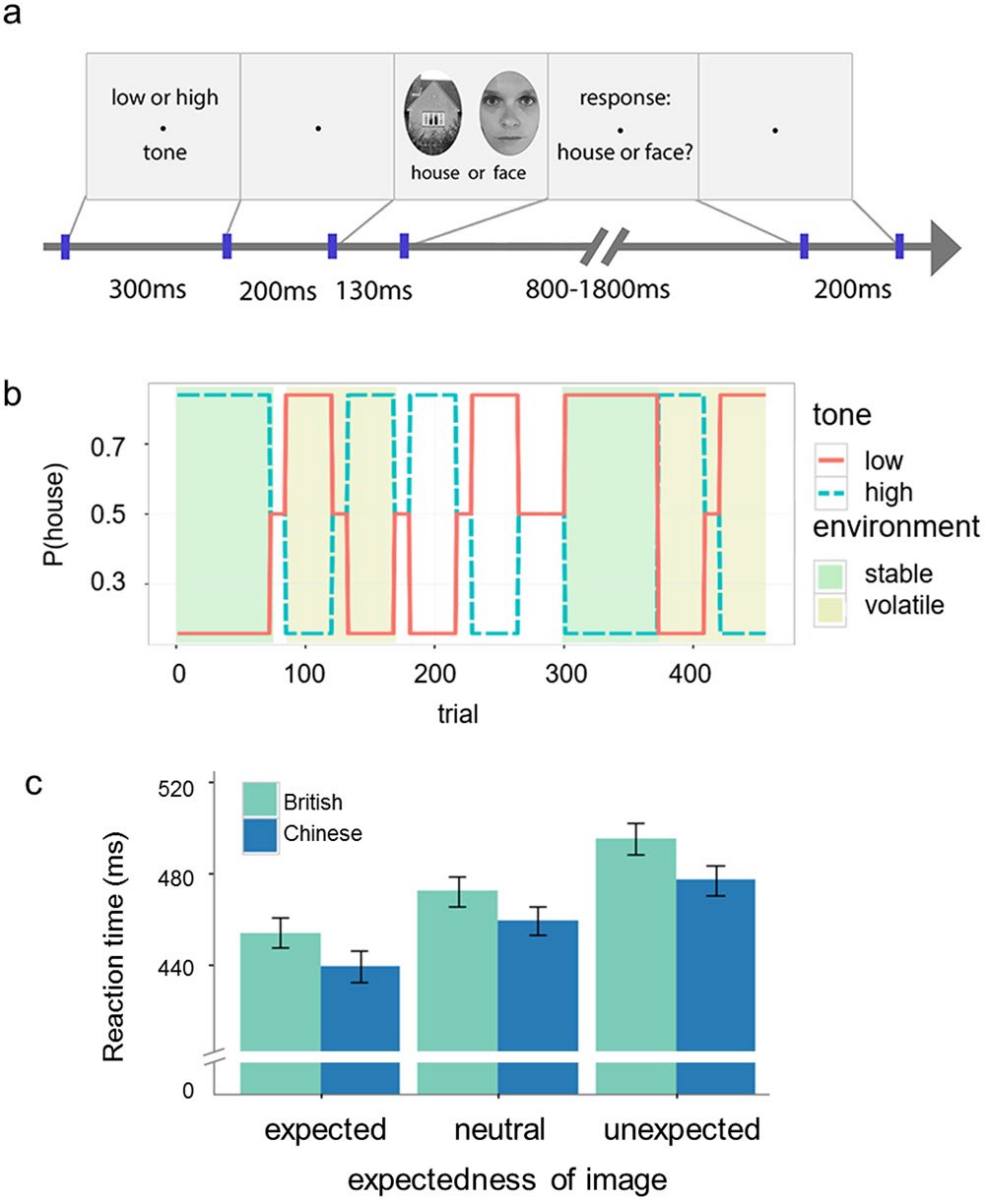
PAL task and behavioural results. (**a**) Shows one example trial. Participants respond to each image and indicate whether it is a face or a house. Example face and house stimuli shown here have no noise added to the images. Please see main text for further details. (**b**) Shows the changing probabilistic associations over the course of the experiment, as well as the stable and volatile trial periods. (**c**) The mean RTs of Chinese and British participants by expectedness of the image. RTs increased linearly in both groups with increasing unexpectedness, consistent with violation of expectations (prediction errors), but there was no main effect or interaction with culture. Error bars represent s.e.m.

On each trial, participants performed binary classification of images of faces and houses. A tone preceding each image was either highly-, non- or weakly-predictive of a given outcome (face or house; Fig. 1a), and these associations changed across time (Fig. 1b). As such, trials were classified according to whether the image was expected, neutral or unexpected. Although we had no specific hypotheses about the effect of culture on processing sensory noise, to optimise the task to reveal the effects of expectations on behaviour we orthogonally varied the uncertainty of each outcome image with three levels of noise (low, medium, high)^21^.

Analysing the RT data revealed robust behavioural effects consistent with a violation of expectations (prediction errors), but no difference between cultures (Fig. 1c). We analysed the RTs for correct trials in a 3 expectedness (unexpected, neutral, and expected) × 3 noise (high, medium, and none) × 2 nationality (Chinese and British) mixed ANOVA in which nationality was a between subject factor. Consistent with previous studies^21^, we found significant main effects for noise (*F*(2,136) = 18.980, *p* < 0.001, partial *η*^2^ = 0.218) and, crucially, expectedness (*F*(2,136) = 80.159, *p* < 0.001, partial *η*^2^ = 0.541). Thus, RTs were longer for increasingly surprising visual stimuli (Fig. 1c). There was no main effect of nationality (*F*(1,68) = 1.045, *p* = 0.310, partial *η*^2^ = 0.015) and no significant interactions between nationality and expectedness (*F*(2,136) = 0.354, *p* = 0.703, partial *η*^2^ = 0.005), noise (*F*(2,136) = 0.700, *p* = 0.498, partial *η*^2^ = 0.010), or expectedness and noise (*F*(4,272) = 1.069, *p* = 0.372, partial *η*^2^ = 0.015).

We also computed log Bayes Factors (logBF_10_) as an additional test of the effects of nationality, see Methods for full details. Bayes Factors provide information with a similar purpose to p-values but are argued to hold advantages^24^. They provide a more stringent assessment of the strength of evidence for an effect, for example p-values of 0.05 to 0.01 typically equate to “anecdotal” evidence in terms of Bayes Factors, while moving from “anecdotal” to “substantial” evidence generally equates to p-values < 0.01^25–27^. Further, they enable statements about an alternative hypothesis, so here can provide evidence for an absence of effect rather than just absence of evidence.

This analysis revealed that the winning model contained only the main effects for noise and expectedness (logBF_10_ = 240.8 ± 0.77%; decisive evidence), and was substantially better (4.07 ± 1.16% times better) than a model also containing a main effect of nationality (logBF_10_ = 239.4 ± 0.86%). The winning model was also decisively better (more than 1300 times better) than all models containing interactions with nationality.

### Temporal context: learning about probabilities and volatility

While the model-agnostic analysis above indicates no effects of culture at the level of gross behaviour (collapsed across all trial types), this analysis throws away temporal information and therefore does not have the sensitivity to detect individual differences in how each participants learn to expect a given outcome across time. Therefore, to investigate learning about probabilistic relationships and volatility across cultures we adopted a participant-specific Bayesian model. The Hierarchical Gaussian Filter (HGF)^18,28^ applied here has been used previously for this same task, to examine context dependence in autism^21^. Please see Lawson *et al*.^21^ and the Methods for full model details.

**Figure 2:**
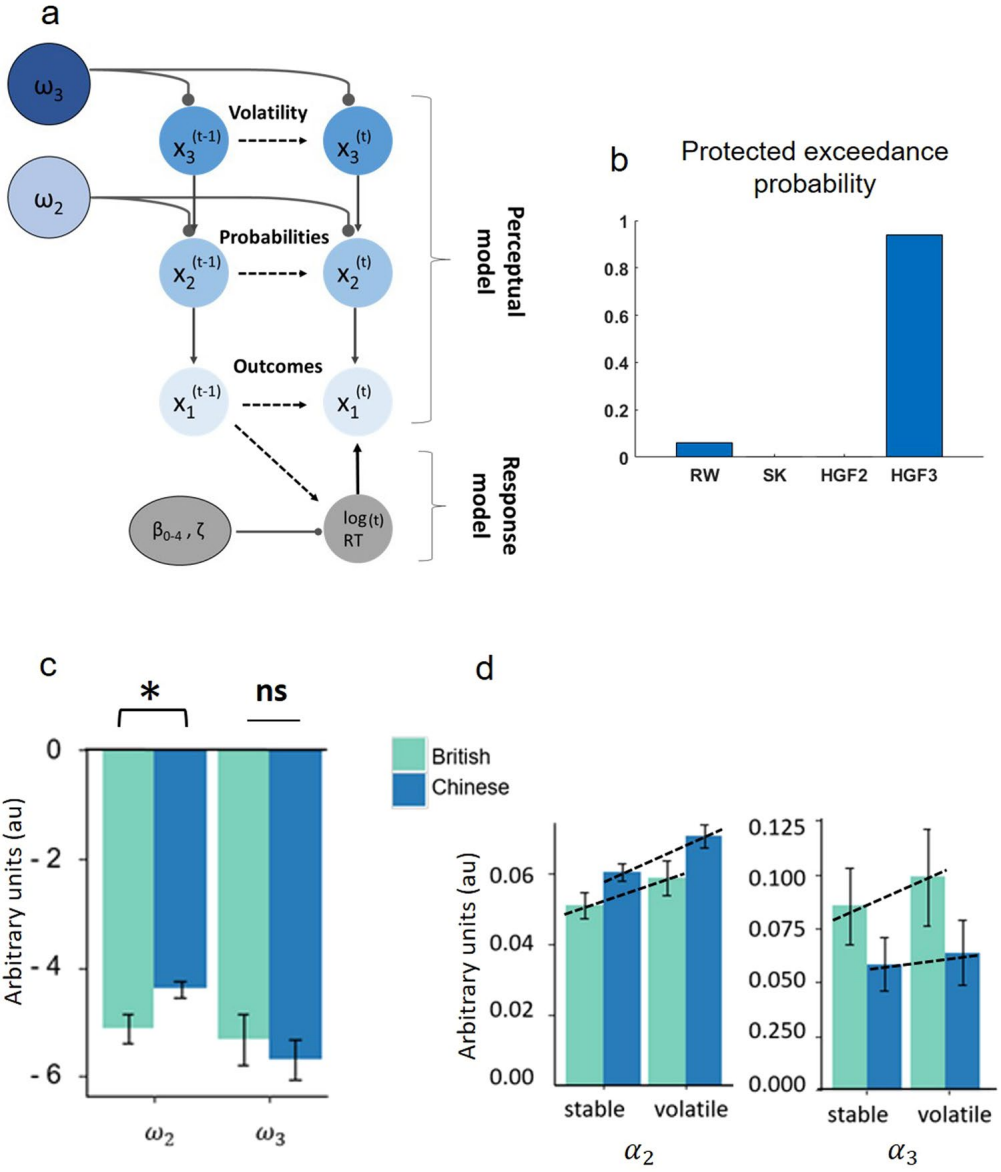
Computational model and model based results for PAL task. (**a**) Shows a schematic of the three level HGF model. See main text and methods for full model details. (**b**) Shows the results of the model comparison. (**c**) Chinese participants tend to have a higher *ω*_2_ than British participants, but there are no cultural differences in *ω*_3_ (**d**) While all participants increase their learning rates in response to volatility, there is no cultural difference in updating either *α*_2_ or *α*_3_ when switching from stable to volatile task phases. rw = Rescorla Wagner, sk = Sutton-K1, HGF 2 = two level HGF, HGF 3 = three level HGF. * denotes group differences with significance p < 0.05. Dotted lines show the linear increase in learning rate between stable and volatile task periods for each group.

Briefly, learning occurs simultaneously on three coupled levels of an uncertainty hierarchy (*x*_1_, *x*_2_, and *x*_3_). Level 1 (*x*_1_) addresses learning about stimulus outcomes (face or house), level 2 (*x*_2_) addresses learning about probabilities (tone-image contingencies), and level 3 (*x*_3_) addresses learning about changes in these probabilities across time (volatility). The HGF allows us to infer each individual’s beliefs about these sources of temporal context (the perceptual model) and map these beliefs on to their observed RTs (the response model; see Fig. 2a).

Before examining the model parameters, we first fit three alternative learning models to the data and compared them to the three level HGF with random-effects Bayesian model selection^29^. Relative to simple reinforcement-learning models with fixed (RW^30^) and dynamic (SK1^31^) learning rates and a two-level HGF in which volatility updates were eliminated, the three-level HGF was the best model for explaining the data (Fig. 2b). This is consistent with a recent application of this exact model and task to study context effects in autism^21^.

The HGF comprises two parts, the response and perceptual models (Fig. 2a), analysed in turn. The response model parameters (β_0_-β_4_, ζ) capture the effect of trial-wise modelled estimates of surprise, uncertainty, volatility and decision noise on (log) reaction times. Full details of the β’s in the response model are explained in the methods. These trial-wise quantities influenced RTs equivalently in Chinese and British participants, as shown by a multivariate analysis of variance (MANOVA) revealing no effect of nationality across all of the response model parameters (Pillai’s Trace = 0.04, *F*(6,64) = 0.448, *p* = 0.844, partial *η*^2^ = 0.04). This is consistent with our model-agnostic analysis of behaviour collapsed across all trials (see results above and Fig. 1c).

The perceptual model parameters (*ω*_2_, *ω*_3_) are the tonic volatilities at their respective levels and indicate, for each individual participant, how they learn and see the world. As such, *ω*_3_ encapsulates the rate at which volatility is estimated to change (i.e. phasic volatility learning rate) and *ω*_2_ captures the rate at which probabilities are estimated to change (i.e. probability learning rate). A MANOVA revealed a trend towards a significant multivariate effect of nationality on the perceptual model parameters (Pillai’s Trace = 0.074, *F*(2,68) = 2.71, *p* = 0.073, partial *η*^2^ = 0.074). Follow up one way ANOVAs revealed no effect of nationality on *ω*_3_ (*F*(1,69) = 0.36, *p* = 0.55, 95% CI: −1.6–0.8), however Chinese participants had a larger *ω*_2_ than British participants (*F*(2,69) = 5.49, *p* = 0.02, 95% CI: 0.1–1.3; Fig. 2c). Larger *ω*_2_ can be understood similarly to a learning rate, and indicates faster updating of beliefs about probabilistic relationships in Chinese participants. An additional Bayes Factor analysis is in general agreement with these results. A model containing an effect of nationality on *ω*_2_ was anecdotally, 2.46 ± 0.00%, better than the null, and a model containing an effect of nationality on *ω*_3_ was substantially, 3.50 ± 0.01%, worse than the null.

These perceptual model parameters also give rise to dynamic learning rates that change trial-to-trial at each level (*α*_2_, *α*_3_; see methods). This enabled us to additionally assess cultural differences in learning as a result of contextual changes from stable periods of the task (where the probabilities remain fixed; dark green boxes Fig. 1b) to volatile phases of the task (where the probabilities switch multiple times; light green boxes Fig. 1b). When the environmental context is volatile people should increase, or update, their learning rate in response to this change in environmental statistics, an effect that has been demonstrated across perceptual^21^, motor^32^, and reward learning tasks^22,33,34^, but not addressed cross-culturally.

To address learning about probabilities (*α*_2_), a repeated measures ANOVA examining task phase (stable, volatile) with a between-subjects factor of nationality, revealed a significant main effect of task phase (*F*(1,69) = 125.09, *p* < 0.001, partial *η*^2^ = 0.64), indicating that across all participants, learning rates increase in response to volatility (Fig. 2d). There was also a significant main effect of nationality (*F*(1,69) = 4.22, *p* = 0.04, partial *η*^2^ = 0.06), indicating that Chinese participants tend to have a larger overall *α*_2_ (a recapitulation of the effect of culture on *ω*_2_). Crucially, there was no interaction between nationality and task phase, suggesting that both Chinese and British nationals update equivalently (*F*(1,69) = 2.52, *p* = 0.12, partial *η*^2^ = 0.03). An additional Bayes Factor analysis also found that the winning model contained the main effects of task phase and nationality (logBF_10_ = 31.7 ± 5.53%). The model won by a margin at the anecdotal level, being 1.11 ± 5.53% times better than a model only containing the main effect of task phase (logBF_10_ = 31.6 ± 0.18%) and 1.68 ± 5.82% times better than a model containing also containing an interaction between task phase and nationality (logBF_10_ = 31.2 ± 1.81%).

An identical analysis for *α*_3_, which addresses learning about volatility rather than probabilities (Fig. 2d), again revealed a significant main effect of task phase (*F*(1,69) = 11.62, *p* = 0.001, partial *η*^2^ = 0.14), but no main effect of nationality (*F*(1,69) = 1.59, *p* = 0.21, partial *η*^2^ = 0.02) or interaction (*F*(1,69) = 2.1, *p* = 0.15, partial *η*^2^ = 0.03). An additional Bayes Factor analysis revealed that the winning model provided strong evidence for the main effect of task phase (logBF_10_ = 3.2 ± 0.34%). This model was anecdotally, 1.07 ± 0.63% times, better than a model containing both the main effect of task phase and the main effect of culture (logBF_10_ = 3.1 ± 0.53%), and substantially, 3.48 ± 1.88% times, better than a model also containing the interaction between task phase and culture (logBF_10_ = 2.0 ± 1.85%).

### Spatial context: tilt illusion

In addition to the learning task described above, which concerns aspects of temporal context in relation to prediction error, we administered a task to formally assess cultural differences in spatial context. The tilt illusion occurs when the orientation of a grating surrounding a central stimulus causes this central stimulus to appear tilted away from its physical orientation. The tilt illusion is an established measure of contextual effects in low level vision and offers a psychophysically robust and biologically informed compliment to previous cross cultural literature examining the effects of background on the perception of^14^ and memory for objects^12^. The tilt illusion task we employed has previously been shown to illicit large inter-individual differences across participants^35^, making it particularly well suited to address potential systematic differences in spatial context sensitivity across cultures.

In keeping with previous research, we quantified the tilt illusion magnitude as the orientation difference between two physically dissimilar stimuli that appeared perceptually equal in the presence of the surrounding context^35^. See Methods for full task details.

**Figure 3:**
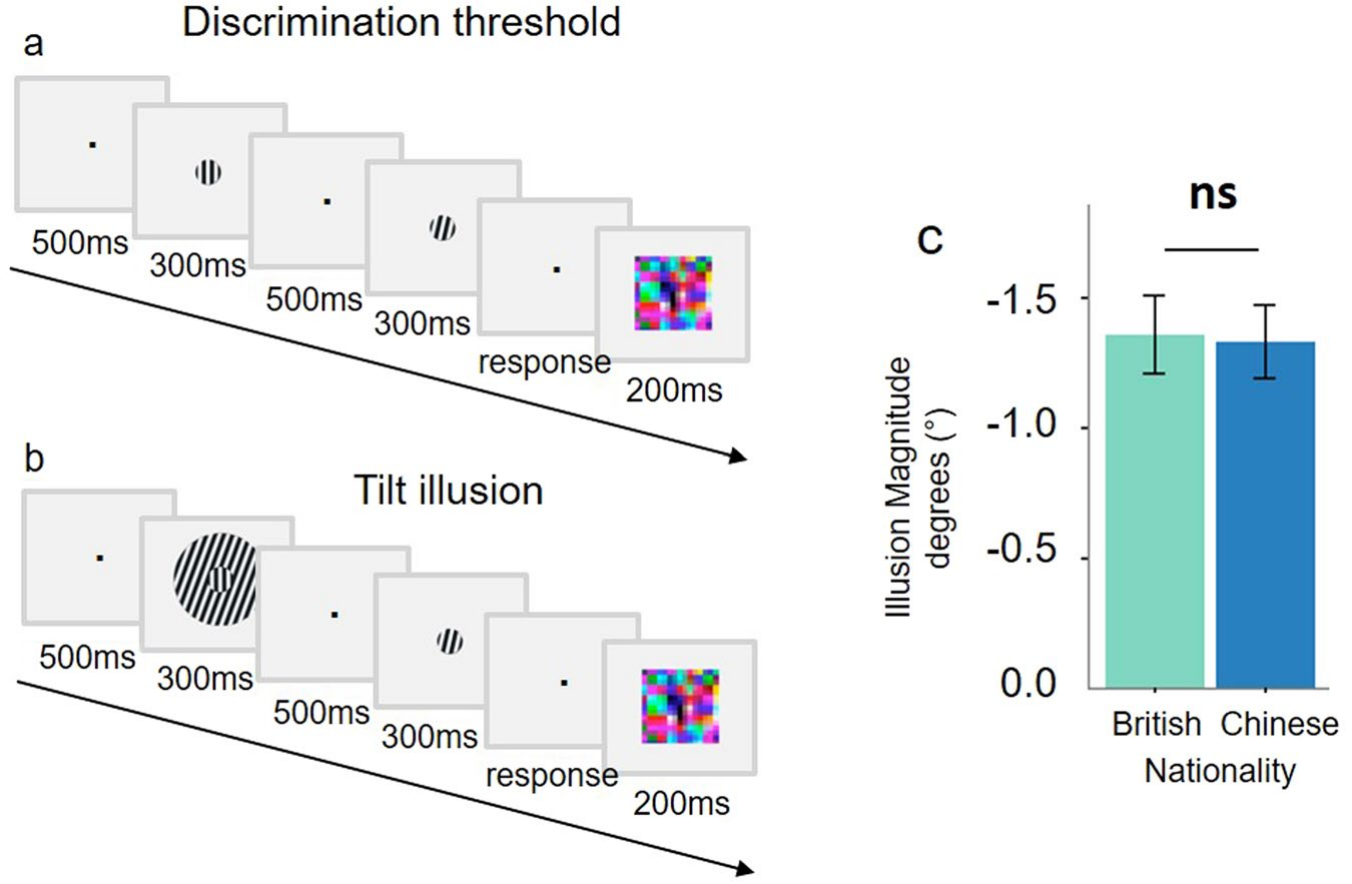
Tilt illusion task and results. (**a**) Shows an example trial from the staircase procedure which first measured orientation discrimination threshold. Participants indicated whether the stimulus in the second interval appeared clockwise or anti-clockwise relative to the stimulus in the first interval. (**b**) Shows a trial in the two alternative forced choice measurement of the tilt illusion magnitude. On each trial, participants indicated whether the central stimulus in the second interval, compared with the central stimulus in the first interval, appeared clockwise or anti-clockwise. (**c**) The tilt illusion magnitude did not differ between cultures. Error bars show s.e.m.

We observed a robust tilt illusion across all participants (Fig. 3c), which was not affected by culture. Tilt illusion magnitudes differed from zero overall (*F*(1,65) = 173.47, *p* < 0.001, *M* = −1.342, *SD* = 0.828, 95%CI: −1.5– −1.1) and in both the British (*F*(1,32) = 82.166, *p* < 0.001, *M* = −1.356, *SD* = 0.859, 95%CI: −1.7– −1.1) and Chinese (*F*(1,32) = 89.129, *p* < 0.001, *M* = −1.328, *SD* = 0.808, 95%CI: −1.6– −1.0) participants. However, there was no cultural difference (*F*(1,64) = 0.01, *p* = 0.9, 95%CI: −0.4–0.4), a result also confirmed by an additional Bayes factor analysis in which a model containing nationality was substantially, 3.94 ± 0.00% times, worse than the null.

### Correlations between measures of context across tasks

We examined whether context effects were domain-general or specific by testing for pairwise correlations between the different measures of context-sensitivity from our two tasks. Tilt illusion magnitude did not correlate with either *ω*_2_ (r(63) = 0.020, p = 0.87) or *ω*_3_ (r(63) = −0.160, p = 0.20), indicating that these assays of temporal and spatial context are relatively domain specific across participants. An additional Bayes factor analysis broadly supports these results, with a model just containing the intercept better predicting the tilt illusion magnitude than a model also containing *ω*_2_. (3.90 ± 0.00% times better; substantial) or than a model also containing *ω*_3_ (1.96 ± 0.00% times better; anecdotal).

## Discussion

The suggestion that there are cultural differences in the extent to which individuals expect change, process prediction errors, and tolerate contradiction is an enduring one^15^, but has never before been robustly assessed using computational metrics of learning or cognitive tasks that assay reflexive behavioural responses to prediction errors. Here we took a computational approach that enabled us to parse components of learning about different forms of temporal context in Chinese and British nationals. We identified robust behavioural effects on RT, consistent with prediction error in the probabilistic associative learning (PAL) task, which did not differ between cultures either in our model-agnostic analysis (Fig. 1c) or in the response model parameters from our computational analysis. However, we found some evidence that culture might affect learning of probabilistic associations in the PAL task, with Chinese individuals learning more quickly about the association between cues and outcomes (Fig. 2c). Contrary to previous psychological reports that East Asians tend to expect more change across time, we found no effect of culture on formal computational metrics of learning about volatility, and while all participants updated their learning in response to sudden fluctuations in environmental contingencies (i.e. switches from stable to volatile periods of the task), this effect did not differ between cultures (Fig. 2d). Finally, in the tilt-illusion task whilst we again identified marked contextual effects of the spatial surround on the perception of the central grating, this spatial context effect did not differ between cultures.

Our finding that behavioural indices of prediction error are similar across Chinese and British nationals is important to understand whether emerging Western biological accounts of neuropsychiatric and neurodevelopmental conditions, that centrally involve prediction error^36–38^, can be transferred wholesale to China or vice versa. For example, recent studies have begun to use the HGF model to interrogate the neurocomputational basis of sensory difficulties in autism^21^ and hallucinations in psychosis^39^. Previous work indicates that prediction errors (or surprises) are experienced differently between East Asian and Western individuals on the basis of *subjective* impacts measured by self-report^8,9^. In contrast, we examined implicit behavioural measures of prediction error (the brief and reflexive slowing down of RT when expectations are violated), and found no cultural difference. One reason for this discrepancy may lie in recent work that has begun to dissociate objective and subjective aspects of prediction errors. For example, trial-wise self-reported fluctuations in wellbeing or happiness are computationally well described as a function or recent rewards vs expectations (i.e. reward prediction errors) but the prediction errors and raw subjective self-reports are reflected in distinct brain regions^40^. Future work could directly compare effects of culture on objective and subjective aspects of prediction error, and one might predict that East Asian nationals will exhibit less subjective impact of prediction errors, but the same magnitude of objective impact on behaviour or neural responses.

Through the HGF, we were also able to model the temporal evolution of behaviour (RTs) in the PAL task and infer individual differences in learning about two different sources of temporal context: probabilistic relationships between cues and outcomes; and changes in these associations across time (i.e. volatility). We found no effect of culture on overall learning about volatility (Fig. 2c) or changes in learning in response to sudden fluctuations in volatility (i.e. switches from stable to volatile periods of the task; Fig. 2d). This contrasts with previous reports that East Asian participants expect more change to occur across time. For example, Chinese students indicate with higher probability that a current couple will break up, or that who enemies will become lovers in later life^23^. When shown a linear trend line, East Asian participants are more likely to predict a reversal in the trend, compared to Western participants who tend to predict further linear increases^23^. Unlike previous studies we explicitly manipulated changeability in this task (i.e., ground truth structure indicated in Fig. 1b) allowing us to objectively measure participant’s estimates of, and responses to, volatility. A recent study applying this task and computational model to autistic, vs neurotypical, adults identified robust differences in how volatility is estimated and related to neural the integrity of the noradrenaline system^21^. Again, the dissociation between subjective reports and objective/neural measurements may be important to reconcile our findings with existing findings in the cross-cultural literature. For example, it has been suggested that cultural differences in holistic processing may be better understood as a “constellation of lay beliefs about the nature of the world, rather than a cognitive style”^15^. Differences between cultures in lay beliefs may become apparent in questionnaires, structured interviews, or open-ended pencil and paper tasks, but need not necessarily constitute a fundamental difference in cognitive or neural processing.

However, our model-based analysis tentatively suggests that there may be cultural differences in probability learning (Fig. 2c). Such effects need to be replicated in future work: previous high-profile cross-cultural findings in other cognitive domains such as memory^12,41^ have shown mixed replicability^42,43^, and the Bayes Factor analyses for the models containing an effect of culture on *ω*_2_ and *α*_2 _were only supported at the “anecdotal” level. Nonetheless it is worth considering the implications of these potential findings. It is a strength of the HGF that probability learning can adapt to the estimated volatility at the higher levels of the model^18^, but equivalent learning about volatility in both Chinese and British participants indicate that the differences in *ω*_2_ are not inherited from the level above. Higher values of *ω*_2_ would produce increased precision-weighting of perceptual belief updates at level 2, making probability updates less reliant on current expectations (over sensory inputs). If the finding that East Asian participants tend towards a larger *ω*_2_ is replicated, this might suggest general effects of culture on the relative weighting of current beliefs relative to new information.

Someone with a large probability learning rate more readily updates their beliefs in the face of new information which could conceivably lead to the pattern of findings in an earlier surprise gift study^9^ – East Asian participants not only report less surprise on receiving an unexpected gift but also a higher estimated probability of receiving an unexpected gift in the first place. However, a general tendency to expect the unexpected would have been captured in the volatility parameters of the HGF, where we observed no effects of culture. It is worth noting that many of the subjective reports of reduced surprise in East Asian participants tend to involve stories or test conditions related to interpersonal interactions, whereas our task involves purely perceptual decision making. Recent research in the domain of social learning suggests that Chinese participants update their beliefs about others’ character traits more readily in light of social feedback^16^. It will be important for future computationally informed studies to examine cross-cultural effects on contingency learning in socially framed tasks^3,44^.

Interestingly, we also found no effects of culture on our spatial context task whilst studies examining spatial context in the Framed Line Test have shown reasonably (although not uniformly^45^) replicable cultural effects comparing East Asian and Western individuals^13,46–49^. In contrast, tasks examining spatial effects on memory show heterogeneous results: with two studies showing more context dependence in East Asians^12,41^, two showing no effect^42,43^ and one showing no effect in one dataset but an effect in a footnoted follow up^50^. Our study is one of a handful examining spatial effects on perception using robust psychophysical methods, although one previous study used a conceptually analogous “rod and frame task” and reported a cultural difference^14^. Whilst the Ebbinghaus illusion shows cultural differences between East Asian and Western participants^51,52^, two studies examining Navon figures do not as well support cultural influence (one showed an effect on RT but not error rate^53^ and one showed no effect^51^). It is plausible that our null result highlights limited cultural effects in some low-level visual tasks that reflect perceptual processing, rather than post-hoc reasoning about a visual stimulus. Future work could compare cultural effects on spatial context across high and low-level visual tasks also test different East Asian populations, for example different groups within China as well as in Japan or South Korea.

Studies with low power have a reduced chance of detecting a true effect, therefore statistical power is an especially important consideration when reporting null findings^54,55^. Here we pre-determined the minimum sample size necessary to ensure that we had sufficient power to detect a difference between groups of similar magnitude to previous reports^21^. In fact, as effect sizes are often inflated in initial reports^56^, we doubled this minimum sample size and accordingly we had >99% power to detect a true difference between groups. We are therefore confident that the absence of differences between cultures reported here are not due to insufficient power and our Bayes Factor analyses provide evidence in favour of the null where appropriate.

In summary, here we combine advanced computational approaches in decision and perceptual neuroscience, with cross-cultural comparisons in traditional psychology, to study the susceptibility to prediction error and the sensitivity to temporal and spatial contexts in different cultures. Across two different tasks and their computational/psychophysical measures, we found that the cross-cultural similarities in objectively measured effects of temporal and spatial context are perhaps more striking than the cross-cultural differences. Given the fast growth of China’s scientific output, identifying robust cross-cultural commonalities and differences in psychological process matters profoundly to interpret scientific findings and additionally has important economic and social implications beyond academia.

## Methods

### Participants

72 participants gave informed consent (36 Chinese and 36 British; both groups balanced for gender) for a study approved by the University College London Ethics Committee (4357/002). All participants were neurologically normal, had no psychiatric history, no hearing difficulties and normal or corrected-to-normal vision. All participants gave written informed consent to take part in this study and were compensated financially for their time. All methods used were in accordance with the relevant guidelines and regulations.

Age did not differ between Chinese (mean 24.2 years +/− standard deviation 2.7) and UK participants (mean 24.7 years +/− 6.7; *F*(1,71) = 0.175, *p* = 0.681). Chinese participants were Chinese nationals who had moved to the UK within the previous two years, of whom a majority (83%) had moved to the UK less than one year before. UK participants were UK nationals who had spent their entire lives in the UK other than ≤1 year abroad.

IQ was assessed using the Cattell Culture Fair Intelligence Test^57^. The groups did not differ significantly on IQ (*F*(1,71) = 3.270, *p* = 0.075; Chinese: *M* = 124.544, *SD* = 7.988, *range* = 102.2–133.8; British: *M* = 120.653, *SD* = 10.146, *range* = 102.2–133.8). We also administered a holism questionnaire developed to assess holistic vs. analytic reasoning^58^. Chinese participants (*M* = 5.403, *SD* = 0.798) had a more holistic thinking style than British participants (*M* = 4.922, *SD* = 0.629; *F*(1,70) = 8.049, *p* = 0.006) using this self-report measure.

### General procedure

To render their culture more salient, participants were tested in pairs of two of the same culture (although with no-shows, 4 Chinese and 8 UK participants were tested alone) and they wrote a short description in their native language about a memorable New Year’s celebration. Participants then completed the two tasks described below, with the order counterbalanced between participants. The tasks were performed on two identical Dell Optiplex 980 Desktop Computers, with Dell 2007FPb 20″ UltraSharp displays (1600 × 1200) with a 4:3 aspect ratio and a refresh rate of 60 Hz.

### Probabilistic associative learning (PAL) task

We implemented a previously described PAL task^21^ to test for cultural modulation of expectation violations (prediction errors) on RTs. As shown in Fig. 1a, on each trial participants heard an auditory stimulus for 300 ms (either a low 330 Hz or high 660 Hz pure tone) followed by a 200 ms interstimulus interval (ISI), and then saw a visual stimulus for 130 ms (either a face or house), before responding by button press if it was a face or house (time for response was randomly generated between 800 and 1800 ms). Finally, participants waited for another 200 ms before the trial ended. The association between the tones and the pictures was either highly (*p* = 0.84), neutral (*p* = 0.5), or weakly (*p* = 0.16) predictive. The probability of one type of visual stimulus, given one type of auditory stimulus, was always equal to one minus the probability of the other visual stimulus, given the same auditory stimulus, thus *p*(face|hightone) = 1 − *p*(house|hightone). Because each block contained the same number of randomly intermixed low and high tone trials, the marginal probabilities of houses and faces were identical before the auditory stimulus was presented, i.e. *p*(face) = *p*(house).

Participants completed three blocks. The first block consisted of 168, the second of 120, and the third of 168 trials, for a total of 456 trials. Within each block periods of highly, neutral, and weakly predicted trials changed pseudo randomly in blocks of 12, 36, or 72 trials. Each period and cue type contained equal numbers of low, medium, and high noise trials.

### Computational analysis of the PAL task

To computationally assess learning about different sources temporal information across cultures we used a widely adopted participant-specific Bayesian model to track the role of distinct forms of uncertainty on behaviour (log RTs). The HGF has been recently applied in several studies of learning under conditions of volatility^19–21,32,39,59–62^. The version of the HGF applied here has been used previously to examine the effect of context dependence in autism^21^. Curtailed model details are provided below to avoid unnecessary repetition, please see Lawson *et al*.^21^ for full model details.

For each participant, the perceptual-model parameters *ω*_2_ and *ω*_3_, and the response model parameters (β0-β4, ζ) were estimated from the trial wise log RT measures by using variational Bayes, as implemented in the HGF toolbox (http://www.translationalneuromodeling.org/tapas/).

#### Perceptual model

The HGF’s perceptual model tracks a participant’s learning of the task’s structure: the trial wise stimulus outcomes at level 1 (*x*_1_), the probabilistic relationship between the tone and the outcome at level 2 (*x*_2_), and the volatility of these relationships at level 3 (*x*_3_). Beliefs are updated trial-to-trial via prediction errors, which evolve as trajectories across time, with dynamic learning rates (the precision weight on the prediction error) at each level (*α*_2_, *α*_3_). Two participant-specific perceptual parameters, *ω*_2_ and *ω*_3_, allow for individual differences in approximate Bayes-optimal learning.

*ω*_3_ determines the rate at which estimates of trial wise phasic volatility are updated. *ω*_2_ captures a tonic (learning) rate at which probabilistic relationships are estimated to change.

#### Response model

The response model captures the mapping from a participant’s trial wise beliefs, i.e. the perceptual model, on to behavioural responses (log RTs). The response model is a simple linear model of the form:

1 $${\rm{l}}{\rm{o}}{\rm{g}}\,R{T}^{(t)}\sim {\mathscr{N}}({\beta }_{0}+{\beta }_{1}\cdot {{\rm{s}}{\rm{u}}{\rm{r}}{\rm{p}}{\rm{r}}{\rm{i}}{\rm{s}}{\rm{e}}}^{(t)}+{\beta }_{2}\cdot {{\rm{u}}{\rm{n}}{\rm{c}}1}^{(t)}+{\beta }_{3}\cdot {\rm{u}}{\rm{n}}{\rm{c}}{2}^{(t)}+{\beta }_{4}\cdot {{\rm{v}}{\rm{o}}{\rm{l}}{\rm{a}}{\rm{t}}{\rm{i}}{\rm{l}}{\rm{i}}{\rm{t}}{\rm{y}}}^{(t)}+\zeta )$$

where *β*_0_ is baseline log RT, *β*_1_ represents surprise about the outcome at the lowest level of the HGF (i.e. outcome prediction error), *β*_2_ and *β*_3_ capture the uncertainty (i.e. variance) of the beliefs at levels 1 and 2 respectively and *β*_4_ is the phasic volatility at level 3. Here ζ captures decision noise. 

### Psychophysical measurement of visual context effect: the tilt illusion

We used a previously validated paradigm^35^ to measure the susceptibility to tilt illusion and quantify cultural differences in the effects of spatial context^63^. In the tilt illusion a central test grating is surrounded by a grating with a different orientation, and this context alters the perceived orientation of the central test stimulus^63,64^. The tilt illusion magnitude is quantified as the orientation difference between two gratings that appeared perceptually equal because of the presence of the surrounding context. We used a temporal two-alternative-forced-choice paradigm with stimuli presented at central fixation. Stimulus parameters are indicated below.

#### Procedure

First, each participant’s orientation discrimination threshold was measured using a two alternative forced choice staircase. Two circular grating stimuli (diameter = 1.5 degrees of visual angle, spatial frequency = 1.5 cycles per degree) were presented one after another and participants indicated by button press whether the second grating was tilted clock- or anticlockwise compared to the first (Fig. 3a). Each stimulus appeared for 300 ms, with a 200 ms interstimulus interval, each trial terminated with a dynamic randomly sampled coloured mask to eliminate afterimages. One stimulus had a fixed orientation of 0° and the orientation of the other was adapted according to a 1-up-2-down staircase, in which an incorrect response increased the difference between the two stimuli by 0.1° on the next trial, whilst after two consecutive correct responses the difference was reduced by 0.1° on the next trial. The procedure stopped after the direction of the staircase had reversed 19 times, at which point we computed the orientation value at which participants were correct 70.71% of the time as the average of the last 13 reversals^65^.

To measure contextual illusion magnitude, two central circular stimuli (diameter = 1.5 degrees of visual angle, spatial frequency = 1.5 cycles per degree) were presented in succession, one with and one without surrounding annular context (diameter = 6 degrees of visual angle). Participants made a forced choice regarding whether the central stimulus in the second interval, compared with the one in the first interval, was rotated clockwise or anticlockwise. Each stimulus appeared for 300 ms, with a 200 ms interstimulus interval, each trial terminated with a dynamic randomly sampled coloured mask to eliminate afterimages. The interval containing the surrounding grating was randomised and counterbalanced across trials. In all trials, one stimulus was fixed (a central grating of 0° with a 20° annular surround), whilst the other stimulus was a grating with no surround in which the orientation varied between trials (Fig. 3b). For the stimulus that varied between trials there were seven possible orientations (o_1_ to o_7_) calculated according to the following formula:

2 $$[\begin{array}{ccc}\begin{array}{cc}{{\rm{o}}}_{1} & {{\rm{o}}}_{2}\end{array} & \begin{array}{cc}{{\rm{o}}}_{3} & {{\rm{o}}}_{4}\end{array} & \begin{array}{cc}{{\rm{o}}}_{5} & \begin{array}{cc}{{\rm{o}}}_{6} & {{\rm{o}}}_{7}\end{array}\end{array}\end{array}]=[\begin{array}{ccc}a & \cdots  & a\end{array}]+1.5s[\begin{array}{ccc}\begin{array}{cc}-3^\circ  & -2^\circ \end{array} & \begin{array}{cc}-1^\circ  & 0^\circ \end{array} & \begin{array}{cc}1^\circ  & \begin{array}{cc}2^\circ  & 3^\circ \end{array}\end{array}\end{array}]$$

in which *a* is the participant’s point of perceptual equality (see below) and *s* is the participant’s orientation discrimination threshold. Each of the seven orientations was presented 18 times in randomized order for a total of 128 trials.

Before measuring the tilt illusion, we measured each participant’s point of perceptual equality. Participants undertook four trials in which they saw two gratings simultaneously on screen (both rotated by 0°, seven visual degrees apart), one of which had a 20° annular surround. Participants were asked to rotate the grating without the surround using button presses in order to match the rotation of the grating with the surround.

### Analysis of the tilt illusion task

We fit each participant’s data from the tilt illusion phase of the experiment with a psychometric function using the Palamedes MATLAB toolbox^66^. The psychometric function is given by:

3 $$\psi (x;a,b,c,d)=c+(1-c-d)F(x;a,b)\,$$

in which *F*(*x*; *a*, *b*)describes the probability of perceiving the two central stimuli (one with and one without surrounding annular context) to have different orientation as a function of their physical difference in orientation (*x*). The parameter *a* reflects the tilt illusion magnitude, and the parameter *b* reflects the orientation discrimination threshold. The tilt illusion magnitude (as opposed to the orientation discrimination threshold) quantifies the susceptibility to spatial context^35,67–72^ and is compared across cultures here.

We fixed both *c* (the probability of guessing the stimulus orientation correctly, independently of it being perceived correctly) and *d* (the probability of making an incorrect response, independently of the perceived stimulus orientation) at 0.02 as this is suggested to introduced less bias than fixing them at 0^73^.

We used the logistic function:

4 $$F(x;a,b)=\frac{1}{(1+\exp (-b(x-a)))}$$

The parameters *a* and *b* were estimated for each participant according to a maximum likelihood criterion. Standard errors for *a* and *b* were calculated by performing a parametric bootstrap based on 1000 simulations. The assumptions about the form of *F*(*x*; *a*, *b*) as well as the fixed values of c and d were tested with goodness-of-fit tests based on 1000 Monte Carlo simulations^66^. Participants were excluded if the goodness-of-fit test failed or if the 68% bootstrapped confidence interval for the illusion magnitude was greater than the range of orientation values (o_1_ to o_7_) used in the trials.

### Statistics, exclusions and power

Frequentist analysis was conducted in R (3.4.1) using the ggplot2, reshape2, and afex packages and MATLAB with the Statistics Toolbox Release 2015b (The MathWorks, Inc., Natick, Massachusetts, United States). In the PAL task, repeated measures analysis of variance (ANOVA) with the between-subjects factor of culture was used to assess group differences across the different conditions in our model-agnostic behavioural analysis. Multivariate analysis of variance (MANOVA; Pillai’s Trace) was used to assess for the multivariate effect of culture across the different HGF model parameters for each participant. Repeated measures ANOVAs, with a between-subjects factor of culture, were used to compare the dynamic learning rates. All follow up post-hoc tests reported throughout are two-tailed. To test for pairwise correlations across the two tasks, we used the Pearson product-moment correlation.

All additional Bayes factor analyses were conducted in R (3.4.1), using the BayesFactor package with the default prior. A Bayes factor is a continuous measure of evidence representing the probability of the observed data in one model compared to another. The winning model was the model with the highest BF_10_ relative to the null hypothesis. The proportional error associated with a BF_10_ is an estimate of the error due to the randomness of the Markov Chain Monte Carlo sampling. When comparing models, the relative success of one model over another was computed by dividing the BF_10_. Values greater than one indicate that a model is better than the comparison. Semantic labels were assigned to the magnitude of these comparisons to aid interpretation, ranging from anecdotal (1–3), to substantial (3–10), to strong (10–30) to decisive (>100; for reference see^74^). When interactions are reported, the model also always includes all the main effects of the interaction.

In the behavioural analysis for the PAL task two Chinese participants were excluded as RTs were unavailable for ≥1 of the nine within-subject conditions. In the tilt illusion task six participants (three Chinese) were excluded from the analysis because their staircase did not converge or goodness-of-fit test showed that their data in the contextual illusion phase was not well described by a logistic function.

To ensure that we had sufficient power to detect a significant behavioural difference between cultures we *a priori* determined our minimum sample size based on the effect size (partial η² = 0.203) for the linear group*expectation interaction previously reported using the same probabilistic associative learning task administered to adults with autism and neurotypical controls^21^. The power calculation was conducted using G Power^75^ 9.1.9.2 and indicated that we required a minimum sample size of 34 participants. With a total sample size of 70 participants we had >99% power to detect effect size of d = 0.5 at α = 0.05.

### Data availability

The datasets associated with this study are available from the corresponding author on reasonable request and in accordance with local ethics rules.
